# Probiotic Microorganisms: A Closer Look

**DOI:** 10.3390/microorganisms5020017

**Published:** 2017-04-08

**Authors:** Julio Villena, Haruki Kitazawa

**Affiliations:** 1 Laboratory of Immunobiotechnology, Reference Centre for Lactobacilli (CERELA-CONICET), Tucuman CP 4000, Argentina; 2 Food and Feed Immunology Group, Laboratory of Animal Products Chemistry, Graduate School of Agricultural Science, Tohoku University, Sendai 980-0845, Japan; 3 Livestock Immunology Unit, International Education and Research Center for Food Agricultural Immunology (CFAI), Graduate School of Agricultural Science, Tohoku University, Sendai 980-0845, Japan

In recent decades; scientists have provided encouraging evidence that probiotic microorganisms are valuable in the prevention and treatment of a variety of diseases. The use of probiotics is expanding dramatically because of our growing understanding of how these beneficial microorganisms exert their effects and which strains are effective for specific conditions.

Several beneficial effects have been attributed to probiotic consumption in clinical trials as well as in vitro and in vivo experimental models. One interesting example is an application of these microorganisms as a non-pharmaceutical approach to help manage human metabolic disorders [1]. In this regard, research from several laboratories around the word has demonstrated that the regular consumption of probiotics is able to improve the serum lipid profile, in particular by reducing serum cholesterol levels. Moreover, scientists have shown that the hypocholesterolaemic effect of probiotics could be explained by different mechanisms that include: decreased absorption of intestinal cholesterol, deconjugation of bile salts; modulation of lipid metabolism through changes in cytokine and adipokine profiles, incorporation or assimilation of cholesterol in the bacterial membrane, and inhibition of the expression of the intestinal cholesterol transporters [1]. More recently, the search for beneficial effects of probiotics on metabolism has moved to the modulation of serum uric acid levels. It is well known that the excessive intake of purine-rich foods such as animal and fish meats that contain high amounts of inosine and its related purines is crucial for the increase of serum uric acid levels. Few reports have investigated the effect of probiotics ingestion on hyperuricemia. In this Special Issue of *Microorganisms*, Yamada and co-workers explored this field. Those authors previously showed that *Lactobacillus gasseri* PA-3 incorporates adenosine and its related purines and that oral treatment with the PA-3 strain diminished adenosine absorption in rats [2]. Here, they investigated whether *L. gasseri* PA-3 also incorporates inosine-monophosphate, inosine, and hypoxanthine, and whether it reduces their absorption in rats. Interestingly, the work reported that the oral administration of *L. gasseri* PA-3 reduced the absorption of the three studied compounds. The PA-3 strain may therefore protect individuals against elevated serum uric acid levels and could have a positive effect in the reduction of hyperuricemia and the prevention of gout [3]. They have successfully produced a functional fermented products based on their studies in Japan.

The expanding knowledge of probiotic effects is also modifying the way in which we see classical functional foods such as yogurt or kefir. *Lactobacillus delbrueckii* subspecies *bulgaricus* and *Streptococcus thermophilus* have generally been used as starters for yogurt production. Yogurt is considered a dairy food carrying viable bacteria with health-promoting effects. In fact, some immunomodulatory properties have been attributed to yogurt. However, as the efficacy of probiotics was discovered to be strain-specific, researchers have questioned whether the beneficial effects of yogurt could be improved by the use of selected *L. delbrueckii* and *S. thermophilus* strains with specific properties [4]. In this regard, it was reported that a yogurt developed with *L. delbrueckii* CRL423 and *S. thermophilus* CRL412, strains previously selected by their immunomodulatory capacities, was able to improve respiratory immunity and protect against *Streptococcus pneumoniae* infection [5]. In this issue Kamiya et al., [6] aimed to evaluate the immunomodulatory activity of two bacterial strains from yogurt *L. delbrueckii* ME-552 and *S. thermophilus* ME-553. By performing in vitro and in vivo experiments, authors investigated the effect of both strains on T cell function. It was shown that the oral administration of the ME553 strain to C57BL/6 mice enhanced the amount of IFN-γ and IL-17 produced by CD4^+^ T cells from Peyer’s patches [6]. These results open the door to interesting future research in order to develop a probiotic yogurt with the capacity to stimulate mucosal immunity.

Probiotics have been used not only to improve immunity but to modulate excessive or unproductive inflammation as well. Thereby, probiotics have found a potential place in the management of inflammatory diseases such as allergies. Intervention studies with probiotics have demonstrated beneficial effects on allergic sensitization and IgE levels, having an impact on eczema, wheezing illnesses and asthma [7]. It is well established that mast cells play a critical role in IgE-mediated allergic diseases, and the degranulation of these cells is important in the pathogenesis of allergy. The study of Harata et al., [8] evaluated the ability of 23 lactobacilli strains, including strains isolated from the human intestine, to regulate degranulation of mast cells. The results suggested that lactobacilli, particularly those from the human intestine, are able to reduce the activation of mast cells in a strain-dependent manner [8].

In addition to the application of probiotics in the prevention or treatment of different diseases, great advances have been made in the understanding of the cellular and molecular mechanisms of probiotic action. Whole-genome sequencing of commensal and probiotic microbes and the study of microbiota using advanced genomic techniques have allowed us to piece together the relationship between the host and bacteria within the complex ecosystems of the human body. In this regard, adhesion of microbes to the mucosal tissues has been considered one of the key initial events in the successful colonization of the gastrointestinal tract. Therefore, there is considerable interest in determining the molecular mechanisms that probiotic bacteria use to colonize their host, and a substantial amount of research has been performed in this filed. As discussed in the review of Nishiyama, Sugiyama and Mukai [9], probiotic lactobacilli exhibit various adhesive properties on mucin and mucin-related carbohydrate chains based on a wide variation of molecular structures. This implies that lactobacilli adapt to the constantly changing intestinal environment of the host, and further suggests that adhesion factors of these microorganisms possess specific binding affinities that allow them to colonize the host and avoid competition with other bacteria. Thus, understanding the interactions between probiotic lactobacilli and mucin is crucial for elucidating the survival strategies of these bacteria in the gut. The review highlights the characteristics of the interactions between lactobacilli and mucin, and concomitantly considers the gastrointestinal structure from a histochemical perspective. Further, biofilm formation has been recently recognized as an important property in the interaction of probiotics with the host. Numerous investigations have verified the beneficial effect of probiotic strains in biofilm form, including increased resistance to temperature, gastric pH and mechanical forces to that of their planktonic counterparts. In addition, the development of new encapsulation technologies, which have exploited the properties of biofilms in the creation of double coated capsules, has given origin to fourth generation probiotics. Up to now, reviews have focused on the detrimental effects of biofilms associated with pathogenic bacteria. On the contrary, the review of Dr. Garcia and co-workers [10], aimed to amalgamate the information describing the biofilms of probiotic strains and their role in probiotic functions. In particular, the review highlights that biofilm formation by probiotic bacteria is considered a beneficial property because it promotes colonization and longer permanence in the mucosa of the host, avoiding colonization by pathogenic bacteria. Moreover, the article reviews the development of probiotics using technology inspired by biofilms.

The science of probiotics has proven that some of these beneficial bacteria exert their beneficial effects on the immune system through several molecules including cell wall, peptidoglycan and exopolysaccharides (EPS) that are able to interact with specific host cell receptors such as pattern recognition receptors. In the review of Laiño et al., [11], the current knowledge of the health-promoting actions of EPS from probiotic bacteria with special focus on their immunoregulatory actions are summarized. In addition, the article describes the studies evaluating the molecular interactions of EPS from probiotics with intestinal epithelial cells, and highlights the role of the pattern recognition receptors and their signaling pathways in the anti-inflammatory capacities of probiotic EPS. A detailed molecular understanding should lead to a more rational use of probiotics in general, and their EPS in particular, as efficient prevention and therapies for specific immune-related disorders in humans and animals.

The articles presented in this Special Issue investigate several aspects of probiotic interaction with the host and their beneficial impact on health. We expect that these articles will stimulate new discussions and investigations to cover the great diversity of probiotics, applications and mechanisms of action that still need to be addressed.
